# The Attitude of the Massachusetts Farmers’ League

**Published:** 1895-11

**Authors:** 


### THE ATTITUDE OF THE MASSACHUSETTS FARMERS' LEAGUE.

We trust that the proposed movement of the Farmers' League in Massachusetts looking to the wiping out of the Tuberculosis Commission and the placing of this work under the direction of the State Board of Health will meet with little support. Admitting all the errors made by this body, there is not in the length and breadth of our land a similar body of men so thoroughly acquainted with every aspect of this subject; none who will profit more, in promoting the best interests of their people, by these errors than they. Their most earnest and bitter opponents have never charged them with dishonesty or deceit, and can only charge errors and blunders made, due to the fact that the work was comparatively ill-understood when pursued on such an extended scale and under circumstances which operated against the accuracy of positive results, proof against which none will dare say that they could have been otherwise. The conscientious devotion to this work, at great personal sacrifice and loss, by members of this Commission is well known to a large number of the profession who have watched this work closely, and we say to Massachusetts that from none can there be greater results obtained than from these trained servants of her people, and we could voice the wish that other States had as valuable a working corps.

